# Impact of ^68^GA-PSMA PET / CT on treatment of patients with recurrent / metastatic high risk prostate cancer - a multicenter study

**DOI:** 10.1590/S1677-5538.IBJU.2017.0632

**Published:** 2018

**Authors:** Aline B. Mattiolli, Allan Santos, Andreia Vicente, Marcelo Queiroz, Diogo Bastos, Daniel Herchenhorn, Miguel Srougi, Fabio A. Peixoto, Lisa Morikawa, João Luiz Fernandes da Silva, Elba Etchebehere

**Affiliations:** 1 Universidade Estadual de Campinas (UNICAMP), Campinas, Brasil;; 2 Medicina Nuclear de Campinas, Campinas, SP, Brasil;; 3 Hospital Sírio - Libanês, São Paulo, SP, Brasil;; 4 Centro de Oncologia de D'Or, Rio de Janeiro, RJ, Brasil;; 5 Grupo Américas Centro de Oncologia Integrado, Rio de Janeiro, RJ, Brasil

**Keywords:** Prostatic Neoplasms, Tomography, X-Ray Computed, Positron-Emission Tomography

## Abstract

**Purpose:**

The purpose of our study was to evaluate the clinical impact of ^68^Ga-PSMA PET / CT in the setting of biochemical recurrence of prostate cancer.

**Materials and Methods:**

We retrospectively evaluated 125 prostate cancer patients submitted to the ^68^Ga-PSMA PET / CT due to biochemical recurrence. The parameters age, Gleason score, PSA levels, and the highest SUVmax were correlated to potential treatment changes. The highest SUVmax values were correlated with age and Gleason score. The median follow-up time was 24 months.

**Results:**

^68^Ga-PSMA PET / CT led to a treatment change in 66 / 104 (63.4%) patients (twenty-one patients were lost to follow-up). There was a significant change of treatment plan in patients with a higher Gleason score (P = 0.0233), higher SUVmax (p = 0.0306) and higher PSA levels (P < 0.0001; median PSA = 2.55 ng / mL).

**Conclusion:**

^68^Ga-PSMA PET / CT in prostate cancer patients with biochemical recurrence has a high impact in patient management.

## INTRODUCTION

Prostate cancer (PC) has the second highest incidence among Brazilian men (non-melanoma cancer has the highest incidence) and in 2018 there will be an estimated 68.220 new cases of prostate cancer (1).

After the initial therapy of patients with high-risk PC (which consists basically of radical prostatectomy (RP) and / or radiotherapy), more than 50% of these patients develop biochemical recurrence, which suggests that conventional diagnostic methods used for initial staging are not able to identify the actual extent of the metastatic involvement (2).

Anatomical imaging such as CT and MRI and metabolic PET / CT imaging with ^11^C-choline or 18F-FDG have somewhat low sensitivities to detect the sites of recurrence.

Multi-parametric MRI with dynamic contrast-enhanced sequences in combination with T2-weighted images is able to detect local recurrence with reported sensitivities as high as 88% (3). Sensitivity varies according to the site of recurrence: 52% in the vesico-ureteral transition; 20% in the retro-vesical space; and 12% to 16% in the bladder (3). WB-MRI has also been shown to assess properly bone metastases with similar performance as bone scintigraphy (4, 5).

^11^C-choline PET / CT presents higher rates of recurrence identification but only when PSA levels are above 2.0 ng / mL (6).

On the other hand, gallium-labeled prostate membrane-specific antigen (^68^Ga-PSMA) PET / CT imaging has been shown to be highly accurate in the setting of biochemical recurrence (including patients with PSA levels below 0.5 ng / mL) (6-10).

The purpose of our study was to evaluate the clinical impact of ^68^Ga-PSMA PET / CT in the work-up of high-risk prostate cancer patients with biochemical recurrence.

## PATIENTS AND METHODS

The Ethics and Research Committee (SLH # 1509238) approved this retrospective multicenter study. The patients were recruited, imaged and followed-up from five different institutions by their respective physicians. Only the ^68^Ga-PSMA PET / CT images were performed in one institution (SLH), in multiple scanners.

Patients with a diagnosis of adenocarcinoma of the prostate underwent ^68^Ga-PSMA PET / CT due to confirmed biochemical recurrence between November 2015 and July 2016. All patients underwent all potential imaging techniques prior to ^68^Ga-PSMA.

Biochemical recurrence was defined according to current guidelines (9, 10). The prostate specific antigen (PSA) levels had to be above 0.2 ng / mL in, at least, two consecutive measurements after radical prostatectomy (RP); a rise ≥ 2 ng / mL above the nadir PSA after radiation therapy (RT) with or without concurrent hormonal therapy.

The intended treatment (pre-^68^Ga-PSMA PET / CT analysis) was defined by each patient's uro-oncologist and/or a tumor board in selected cases. Treatments consisted of one or more of the following: 85.7% radical prostatectomy with lymph node dissection (RP), 49.2% RT, 0.1% chemotherapy, and 34% secondary hormone therapy.

### Conventional and metabolic images prior to ^68^Ga-PSMA PET / CT

All patients with biochemical recurrence and negative conventional images were recruited. Conventional images consisted of pelvic ultrasound, bone scintigraphy (all of them with SPECT / CT using ^99m^Tc-MDP), pelvic MR and CT of the abdomen. Some patients were also submitted to metabolic images, which consisted of PET / CT scans performed with ^18^F-FDG, ^11^C-coline and / or ^18^F-Fluoride PET / CT. The median time interval between performing ^68^Ga- -PSMA PET / CT imaging after all negative conventional imaging was 7.1 days (1 – 180 days). There was only one patient that performed a CT scan 6 months prior to the ^68^Ga-PSMA PET / CT although all of his other conventional images (bone scan and pelvic MR) were acquired less than 10 days prior to the ^68^Ga-PSMA PET / CT scan.

### ^68^Ga-PSMA PET / CT imaging

All patients underwent a true whole-body PET / CT acquisition (Siemens Biograph, Siemens ^®^ Healthcare, USA) 60 minutes after intravenous injection of 185 MBq of ^68^Ga-PSMA (grupoRPH ^®^, Brazil). PET scans were acquired in three-dimensional mode with 4 minutes per bed position. To increase lesion detection, delayed images 3 hours after injection were acquired. CT parameters included 5 mm axial reconstruction and 120 kV or dose care kV tube voltage (11).

### Interpretation and Quantification

Two Nuclear Medicine physicians with over 12 years of experience with PET / CT blindly interpreted all ^68^Ga-PSMA PET / CT images. Images were evaluated for detection of local recurrence, locoregional and extra-pelvic lymph node metastases, bone metastases and visceral metastases. Furthermore, quantitative interpretation was performed on all ^68^Ga-PSMA PET / CT images evaluating the highest SUVmax.

### Evaluation of Change in Patient Management

Classification of change in patient management after the ^68^Ga-PSMA PET / CT study was based on the actual clinical outcome of the patient.

Changes were classified as: positive, negative or unknown. Positive changes occurred when the intention to treat was altered by adding or suspending a previously defined treatment strategy. On the other hand, a change in treatment was considered negative when the ^68^Ga-PSMA PET / CT did not add relevant information capable of changing the previously established intention to treat.

^68^Ga-PSMA PET / CT positive findings were only confirmed with biopsy in selected cases. The majority of the patients had confirmed ^68^Ga-PSMA PET / CT positive findings with follow-up; after proper therapy implementation there was a drop in PSA levels.

#### Statistical analyses

We evaluated the impact of ^68^Ga-PSMA PET / CT on patient management in terms of change in treatment strategy, considering changes in treatment modality (for example, when surgery was switched to RT), intention to treat (curative versus palliative) and within the same modality (ex.: changes in RT target volume). When ^68^Ga-PSMA PET / CT findings did not change the therapeutic approach, the impact was considered null.

Exploratory data analysis was performed through summary measures (mean, standard deviation, minimum, median, maximum, frequency and percentage). The correlation between the numerical variables was performed using the Spearman coefficient.

The parameters age, Gleason score, PSA levels immediately prior to PSMA, and highest SUV (SUVmax) were correlated to potential treatment change and the types of treatment. The highest SUVmax values were correlated with age, Gleason score, duration of cancer, initial PSA levels and PSA levels immediately prior to PSMA. The groups were compared by multivariable analysis through the Mann-Whitney test. The level of significance was 5%.

The SAS System for Windows (Statistical Analysis System), version 9.4. (SAS Institute Inc, Cary, NC, USA) was used for analysis.

## RESULTS

We evaluated 125 male patients, mean age = 69.0 years (Table-1). The mean follow-up period was 24 months and three patients died during follow-up. While biochemical recurrence is defined as PSA rise in 2 consecutive measurements, the PSA values described in Table-1 are only the last values obtained immediately prior to the ^68^Ga- -PSMA PET acquisition.

**Table 1:** Characteristics of patients submitted to 68Ga-PSMA PET/CT.

	Mean	Median	SD	Min	Max
**Biochemical Recurrence (n = 126)**					
Age (years)	68.7	68	8.9	43	89
Gleason score	7.7	8	1.3	6	10
Duration of cancer (months)	91.4	76	65.3	14	309
Initial PSA levels (ng/mL)	12.5	7.0	13.4	0.81	69
Time between PSMA and RP (months)	76.3	61.2	65.6	2.3	273
PSA levels (ng/mL)	26.2	1.8	164.7	0.003	395
Time between PSMA and cancer diagnosis (months)	78.5	63.5	65.1	3.1	298
Highest SUVmax	13	8.2	14.1	1.4	79.4

**Figure 1:**
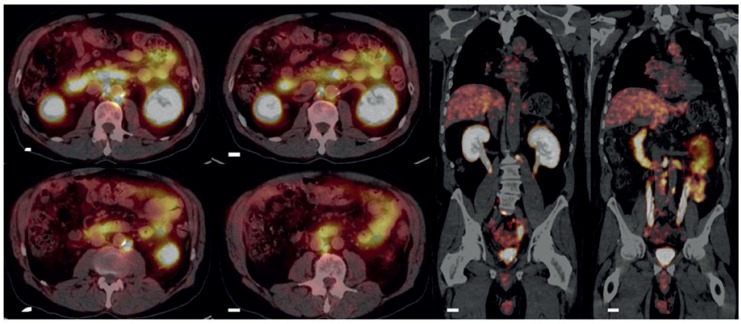
Example of patient submitted to ^68^Ga-PSMA PET/CT due to biochemical recurrence. The prior CT images and MRI performed were negative for metastases. The ^68^Ga-PSMA PET/CT images demonstrate normal size lymph nodes that are avid for ^68^Ga-pSMA.

**PSMA** = ^68^Ga-PSMA PET/CT; **RP** = radical prostatectomy; **PSA** = prostate specific antigen; **RP** = radical prostatectomy;

Among these 125 patients, 80 (64%) had a positive ^68^Ga-PSMA PET / CT. Local recurrence was detected in 30.9%, loco-regional lymph node metastases in 44.4%, extra-pelvic abdominal lymph node metastases in 12.5%, thoracic lymph nodes in 17.5%, visceral metastases in 13.6% and bone lesions 29.6% of patients. (Figure-1).

We were able to evaluate change of management in 104 patients (21 patients were lost to follow-up). Among these 104 patients, 66 (63.4%) patients underwent a change of management after the result of the ^68^Ga-PSMA PET / CT.

^68^Ga-PSMA PET / CT results were positive in 69 patients and a change of management occurred in 85.5% of them (n = 59). Among these 59 patients in which the ^68^Ga-PSMA PET / CT results were positive results were due solely to local recurrence in 11 patients, loco-regional lymph node metastases in 20 patients, visceral metastases in 11 patients and bone metastases in 17 patients.

Treatment was switched to one or more of the following: surgery in 17 patients, chemotherapy in 2 patients, radiotherapy in 23 patients, secondary hormonal therapy in 45 patients and other treatments in 3 patients. In patients with local recurrence, management was altered to one or more of the following: surgical treatment (n = 7), radiotherapy (n = 5) and hormone therapy (n = 5). In patients diagnosed with loco-regional lymph node metastases management was changed to one or more of the following: surgery (n = 7), radiotherapy (n = 8), hormone therapy (n = 9) and chemotherapy (n = 1). Patients diagnosed with visceral metastases were submitted to one or more of the following (considering that the patient also had lymph node metastases as well): hormone therapy (n = 10), radiotherapy (n = 3) and chemotherapy (n = 1). All patients only with bone metastases were submitted to hormone therapy.

On the other hand, ^68^Ga-PSMA PET / CT studies were negative in 35 / 104 patients and there was no change of management in 80% of them (28 / 35 patients).

There was a significant change of treatment plan in older patients (P = 0.0326; median = 69.0 years), patients with a higher Gleason score (P = 0.0233; Gleason = 8.0), higher SUVmax (p = 0.0306; median SUVmax = 8.9) and higher PSA levels (immediately prior to ^68^Ga-PSMA PET / CT) (P < 0.0001; median PSA = 2.55 ng / mL).

Surgery was significantly more likely to occur in younger patients (P = 0.0429) and patients with lower SUVmax values (P = 0.0071).

Higher SUVmax values were associated with higher PSA levels immediately prior to ^68^Ga-PSMA PET / CT (p = 0.38260; P = 0.0005) and older age (p = 0.28423; P = 0.0106) (Table-2).

**Table 2:** Correlation of the highest SUVmax values in patients that underwent ^68^Ga-PSMA PET/CT with the variables age, Gleason score, Duration of cancer, Initial PSA levels and PSA levels immediately prior to PSMA.

	ρ	*P*
Age	0.284	**0.0106**
Gleason score	-0.035	0.7766
Duration of cancer	0.184	0.1006
Initial PSA levels	-0.089	0.5471
PSA levels prior to PSMA	0.382	**0.0005**

Furthermore, higher PSA levels (obtained immediately prior to ^68^Ga-PSMA PET / CT) were significantly (P = 0.0084) associated with the use of chemotherapy (Table-3).

**Table 3:** Patient and ^68^Ga-pSMA PET/CT characteristics in comparison to treatment types (surgery, radiotherapy, etc) and its impact in patient management.

Variables	Surgery	Chemo	RT	HT	Change in management
(P-values)					
Age	**0.0429**	0.1279	0.5983	0.0897	**0.0326**
Gleason score	0.8254	0.2540	0.8884	0.4363	**0.0233**
PSA levels * (ng/mL)	0.6039	**0.0084**	0.2623	0.8808	**<0.0001**
Highest SUVmax	**0.0071**	0.8255	0.1578	0.3569	**0.0306**

* Immediately prior to ^68^Ga-PSMA PET/CT; **NA =** data not available; **Chemo =** chemotherapy; **RT =** radiotherapy; **HT =** secondary hormone therapy

A median PSA level of 3.0 ng / mL was associated with a positive ^68^Ga-PSMA PET / CT study while negative ^68^Ga-PSMA PET / CT studies were noted with a median PSA level of 0.6 ng / mL. (Tables 4 and 5).

**Table 4:** Serum PSA levels obtained immediately prior to ^68^Ga-PSMA PET/CT results.

PSA levels (ng/mL)					
	Median	Mean	SD	Min	Max
Positive	3.0	17.7	56.1	0.003	395.0
Negative	0.6	1.3	1.6	0.1	5.2

**Table 5:** ^68^Ga-PSMA PET/CT results according to serum PSA levels (ng/mL) immediately prior to scan.

PSA levels (ng/dL)	Positive	Negative
<0.5	9 (30%)	21 (70%)
0.5 – 1.0	8 (50%)	8 (50%)
≥ 1.1	63 (82%)	14 (18%)

## DISCUSSION

To our knowledge, this is the first Brazilian study to address the clinical impact of ^68^Ga-PSMA PET / CT in prostate cancer patients. Impressively, change in management occurred in 67% of the patients that underwent the study in the setting of biochemical recurrence. Considering only the number of patients in whom ^68^Ga-PSMA PET / CT results were positive, the potential change of management occurred in 85.5%. Similarly, a recent study found a change in patient management in 53% of patients with biochemical recurrence (12).

We found slightly lower detection rates of recurrence and metastases with ^68^Ga-PSMA PET / CT (64%) when compared to other authors. The sensitivity to detect lesions with ^68^Ga-PSMA PET / CT directly correlates with the PSA levels. Our patient population in which the ^68^Ga-PSMA PET / CT results were negative had median PSA levels of 0.6 ng / mL. For example, Eiber et al. detected lesions in 89.5% of the patients; however, unlike our patient population, 50% of their patients had PSA ≥ 2 ng / mL (8). In another study, ^68^Ga-PSMA PET / CT was positive in 71% when PSA levels were 0.5 – 2.0 ng / mL and in 88% with PSA levels above 2.0 ng / mL (6).

We believe that it is important to understand which variables might interfere with the ^68^Ga-PSMA PET / CT findings. There was a direct and significant correlation between SUVmax and patient age. This could be explained by the fact that higher PSMA expression in prostate cancer tumor cells occur in patients submitted to antihormonal treatment (8, 13, 14) who are usually older, with a higher comorbidity burden and hence for whom surgery is not indicated (15). It seems that higher SUVmax values are associated with higher tumor burden. Consequently, patients with higher SUVmax values are less likely to undergo surgery, as higher SUVmax values are associated with extensive disease and should be switched to systemic therapy.

^68^Ga-PSMA PET / CT results led to considerable treatment changes in older patients, or those with higher Gleason scores, higher PSA levels or higher SUVmax values. Patients with lower SUV-max values were notably younger and prone to undergo salvage surgery, while patients with high SUVmax values underwent chemotherapy, RT or secondary hormonal therapy. We did not find correlation between SUVmax values and the Gleason score, as has been demonstrated, for example, by Verburg et al. (16).

The limitations of our investigation were its retrospective nature, the small number of patients studied and the impossibility to analyze the PSA doubling time. Furthermore, we did not correlate change in patient management with long-term survival. Despite these limitations, our findings are in accordance with the literature and show the potential of ^68^Ga-PSMA PET / CT in altering management, especially for allowing changes in radiation field or indication or not of local therapies. Prospective randomized studies are urgently needed to evaluate its impact on survival and allowing the routine use of this promising imaging agent.

^68^Ga-PSMA PET / CT was able to identify metastases and local recurrence in 64% of patients with negative conventional imaging studies. Remarkably, ^68^Ga-PSMA PET / CT detected a local recurrence with an SUVmax = 3.8 even with rising consecutive PSA levels at a minimum of 0.003 ng / mL. Although there is no justification to encourage performing ^68^Ga-PSMA PET / CT at such low levels of PSA, ^68^Ga-PSMA PET / CT may have a tremendous impact in reducing costs of the public health care system in our country by avoiding unnecessary and futile conventional imaging at low PSA levels. A recent publication has demonstrated that ^68^Ga-PSMA PET / CT is more beneficial as an initial imaging study when work-up is necessary compared to bone scan (17).

## CONCLUSIONS

^68^Ga-PSMA PET / CT has a tremendous impact in the management of prostate cancer with biochemical recurrence. This is a first step towards widespread implementation and applicability of this radiotracer in our country due to its potential cost reduction in our health care system by avoiding unnecessary and futile conventional imaging at low PSA levels.

## ABBREVIATIONS

PC =: prostate cancer
RP =: radical prostatectomy
PET / CT =: positron emission computed tomography
TRU =: transrectal ultrasound
MRI =: magnetic resonance imaging
RT =: radiotherapy
PSA =: prostate specific antigen
G =: Gleason score
SUVmax =: highest Standardized Uptake Value
